# Retraction: One-year outcome and survival analysis of deferred ventricular septal repair in cardiogenic shock supported with mechanical circulatory support

**DOI:** 10.1371/journal.pone.0273165

**Published:** 2022-08-11

**Authors:** The PLOS ONE Editors

After this article [1] was published, the corresponding author requested its retraction because the treatment was incorrectly reported for the five patients listed as having received LVAD. The author stated that these patients should have been in the IABP group, and that they identified errors and evidence of data falsification in the affected patients’ data records.

An official of Rawalpindi Institute of Cardiology contacted PLOS and requested this article’s retraction. They stated that LVADs (Heartmate II Abbott, USA) were not used at Rawalpindi Institute of Cardiology or Mega Medical Complex at the time of the study and noted additional concerns about the validity and authenticity of the patient data in this article [1]. The official also raised that the author did not receive ethics approval from Rawalpindi Institute of Cardiology, and that the Mega Medical Complex did not have an ethics oversight mechanism in place to the best of their knowledge. Hence, the ethics statement referring to Mega Medical Complex approval, ‘written, informed consent was waived off by the review board’ is in question.

In light of the above issues, the *PLOS ONE* Editors retract this article.

FY, AM, MUF, AK, MS, HN, GR, ASR, MU, and SK either did not respond or could not be reached. JM did not respond to the final retraction decision.
